# Microstructural Damage Evolution and Interfacial Failure Mechanism of NC-UHPC Composites Under Seawater Wet–Dry Cycling

**DOI:** 10.3390/ma19081535

**Published:** 2026-04-11

**Authors:** Zhu Wei, Yubin Zheng, Lili Jin, Weiwei Zhu, Yang Yang, Xiaoli Xie

**Affiliations:** 1 School of Municipal Construction and Transportation, Guangxi Polytechnic of Construction, Nanning 530007, China; 18977980815@163.com (Z.W.); jinlili198711@foxmail.com (L.J.); 2 School of Civil Engineering, Guangxi Polytechnic of Construction, Nanning 530007, China; 15060120196@163.com (Y.Z.);; 3 Guangxi Key Laboratory of Advanced Structural Materials and Carbon Neutralization, School of Materials and Environment, Guangxi Engineering Research Center for Advanced Materials and Intelligent Manufacturing, Guangxi Minzu University, Nanning 530105, China; zhuww1230@gxmzu.edu.cn

**Keywords:** normal concrete, ultra-high performance concrete, seawater wet–dry cycling, overlay transition zone, interfacial bond strength, pore evolution

## Abstract

**Highlights:**

- The bond strength increased by 13.53% after 30 wet–dry cycles, then decreased by 41.55% after 90 cycles.
- The damage severity was highest in NC, intermediate in OTZ, and lowest in UHPC under seawater wet–dry cycling.
- The XCT-MIP coupling enables multi-scale tracking of corrosion damage evolution.

**Abstract:**

Composite specimens of normal concrete (NC) and ultra-high performance concrete (UHPC) in marine tidal zones are susceptible to coupled physico-chemical degradation under seawater wet–dry cycling; however, the microscopic damage-evolution mechanisms within the NC/overlay transition zone (OTZ)/UHPC three-phase region remain unclear. In this study, accelerated erosion was conducted using 10-fold concentrated artificial seawater under 0, 30, 60, and 90 wet–dry cycles. The X-ray computed tomography, mercury intrusion porosimetry, backscattered electron imaging coupled with energy dispersive X-ray spectroscopy and slant shear tests were employed to systematically investigate the macroscopic bonding performance and microscopic structural damage of NC-UHPC composites. The results show that the interfacial bond strength initially increases and then declines, exhibiting a 13.53% improvement after 30 wet–dry cycles and a sharp 41.55% decrease after 90 cycles compared with that after 60 cycles. The damage severity was the highest in NC, intermediate in OTZ, and lowest in UHPC. The gas-rich pore region within the OTZ provides a stress-buffering effect during the early stage of corrosion. After 90 wet–dry cycles, the total porosity increased by 0.14%, with external porosity increasing by 0.21% and internal porosity decreasing by 0.07%, indicating a pore-structure reconfiguration characterized by micropore coalescence and an increased proportion of macropores. These findings clarify the damage process associated with seawater erosion, pore expansion, and interfacial failure, providing theoretical support for the repair design and durability assessment of marine concrete structures.

## 1. Introduction

**Figure 1:**
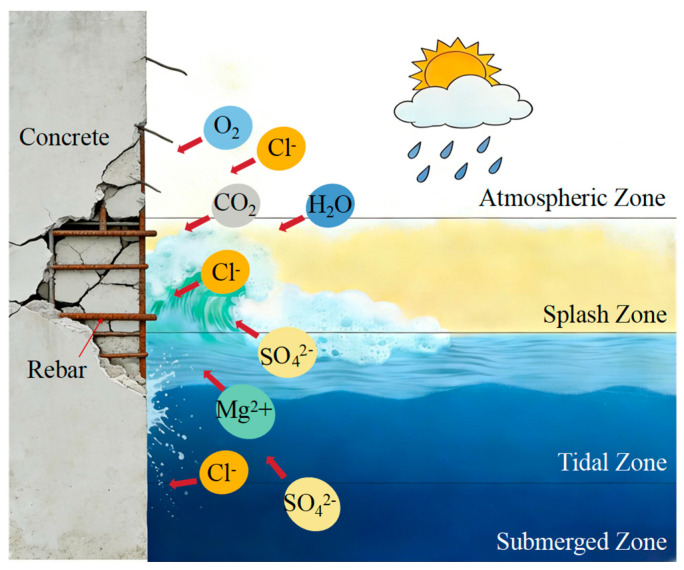
Schematic illustration of marine exposure zones for hydraulic concrete structures in the wet–dry cycling zone.

The coupled effects of chloride ions, sulfate ions, and wet–dry cycles in marine environments are among the primary causes of durability degradation and even failure of concrete structures [1,2,3,4]. Hydraulic concrete structures in coastal and offshore areas can generally be divided into three exposure zones according to their location (Figure 1): the atmospheric salt-spray zone, the splash and tidal zone subjected to alternating wetting and drying, and the underwater zone permanently immersed in seawater. Among them, concrete structures in the tidal zone and splash zone are exposed to the combined action of wet–dry cycles and aggressive ions, such as chlorides and sulfates. Therefore, the wet–dry cycling zone is often the region most vulnerable to steel corrosion in concrete structures [5], which further accelerates the deterioration of their mechanical performance [6]. Normal concrete (NC), owing to its relatively high porosity and limited resistance to marine erosion, is susceptible to corrosion-induced deterioration when exposed to marine tidal zones, thereby significantly shortening the service life of structures [7,8,9]. Ultra-high performance concrete (UHPC), characterized by high strength, low permeability, and excellent resistance to aggressive environments, is widely used for the repair and reinforcement of marine concrete structures. The interfacial bonding performance of the overlay transition zone (OTZ) between UHPC overlays and NC substrates directly governs the overall load-bearing capacity and durability of the resulting composite specimens [10,11,12,13,14].

Extensive studies have investigated the interfacial bonding performance of NC-UHPC composites. Aaleti and Sritharan [15] investigated the shear resistance of UHPC and NC interfaces and identified interface roughness as a key determinant of bond strength. Splitting tensile tests further showed that curing conditions, substrate moisture content, and UHPC age significantly influence the tensile performance of NC-UHPC interfaces [16,17]. Moreover, Javidmehr et al. [18], Santos et al. [19], and Xu et al. [20] reported that interfacial bonding is controlled by multiple factors, including substrate roughness, moisture conditions, adhesive type, and strength compatibility. At the macroscopic scale, surface treatments (e.g., roughening and high-pressure water jetting) enhance mechanical interlocking forces, thereby improving interfacial shear strength [21,22,23]. At the microscopic scale, delayed hydration of unhydrated cement particles in UHPC and the ingress of hydration products into pores of the NC matrix are considered as the key mechanisms underlying chemical bonding [24,25]. However, under marine wet–dry cycles, aggressive ions (e.g., chlorides and sulfates) can preferentially penetrate the porous interfacial region, promoting deterioration of hydration products and reducing bond strength [26,27,28]. As the weak link between NC and UHPC, the microstructure and damage evolution of OTZ directly influence the long-term stability of interfacial adhesion [24,29]. Nevertheless, most existing studies focus on damage characteristics within individual regions [30,31], and systematic, quantitative comparisons of damage evolution across the NC/OTZ/UHPC three-phase region under marine wet–dry cycles remain limited.

To address the durability of NC-UHPC in corrosive environments, Liu et al. [29] investigated the degradation of interfacial bonding under seawater wet–dry cycles and freeze–thaw cycles, showing that a combined interface treatment (beveling + grooving) provides better durability retention than single treatments. Using in situ X-ray computed tomography (XCT), Luo et al. [32] revealed the damage evolution of NC-UHPC composite under salt freeze–thaw cycles and attributed the higher damage tolerance of the OTZ to its higher initial porosity, which offers buffering space for frost-heave pressure. Liu et al. [33] studied UHPC under long-term seawater immersion and observed a three-stage “strengthening–damage–repair” evolution in strength, associated with continued hydration of unhydrated cement particles and subsequent microcrack initiation. Wet–dry cycles, as a typical marine exposure condition, accelerate concrete pore-structure reconstruction and microcrack propagation through periodic ion enrichment, crystallization-induced expansion, and erosion redistribution [34,35]. During drying, salts concentrate, and subsequent rewetting promotes redistribution within the concrete, thereby increasing ionic transport and damage accumulation [36]. The resulting crystalline salts can partially fill cracks but also generate substantial expansive stresses, further driving crack growth [37,38,39]. However, in NC-UHPC composite systems, pronounced differences in porosity, permeability, and elastic modulus among the NC matrix, OTZ, and UHPC lead to heterogeneous responses under the wet–dry cycling [30]. Although UHPC is widely recognized for its low permeability, the region-resolved, quantitative linkage between the pore-structure evolution and interfacial bond degradation across the NC/OTZ/UHPC three-phase system remains insufficiently understood.

For corrosion-damage characterization, traditional scanning electron microscopy and mercury intrusion porosimetry (MIP) can quantify pore-structure features; however, they cannot readily enable dynamic tracking of damage evolution in the same specimen [40,41]. In recent years, XCT has been widely applied to cement-based materials [42,43,44] and enables non-destructive visualization of crack initiation and propagation. Moreover, multiscale porosity assessment [45], pore fractal-dimension analysis [46,47], and identification of OTZ [48] provide valuable tools for multi-scale damage evaluation. Nevertheless, studies integrating XCT with MIP-derived pore-size distributions to elucidate the linkage between macroscopic interfacial adhesion degradation and microscopic pore-structure evolution in NC-UHPC composites under seawater wet–dry cycles remain scarce.

To bridge these gaps, in this study, 10-fold concentrated artificial seawater was used as the erosion medium and wet–dry cycling tests with 0, 30, 60, and 90 cycles were conducted. XCT, combined with MIP, backscattered electron imaging coupled with energy dispersive X-ray spectroscopy (BSE-EDS), slant shear tests, and mass-loss measurements, was used to systematically track microstructural evolution and damage development in the NC, OTZ, and UHPC regions. The results are expected to provide theoretical support and practical guidance for durability-oriented design of UHPC-repaired marine concrete structures by identifying the weak region in the NC/OTZ/UHPC system, clarifying the evolution of interfacial bond deterioration under the wet–dry cycling, and establishing a multi-scale relationship between pore-structure evolution and interfacial degradation.

## 2. Materials and Methods

### 2.1. Raw Materials

The cementitious materials used for NC consisted of ordinary Portland cement (P·II52.5, Fusui Xinning Conch Cement Co., Ltd., Chongzuo, China), ground granulated blast-furnace slag powder (Guangxi Xintumao Technology Co., Ltd., Nanning, China), and limestone powder (Guangxi Xintumao Technology Co., Ltd., Nanning, China). Their chemical compositions and physical properties are listed in Table 1. Manufactured sand was used as the fine aggregate (apparent density: 2720 kg/m^3^; maximum particle size: 5 mm; fineness modulus: 2.78, Nanning Tongdasheng Concrete Co., Ltd., Nanning, China). The coarse aggregate was limestone crushed stone (apparent density: 2710 kg/m^3^; maximum particle size: 25 mm, Nanning Tongdasheng Concrete Co., Ltd., Nanning, China). The polycarboxylate-based high-performance water reducer (No. 1, Guangxi Xingdu Construction Technology Co., Ltd., Nanning, China with a solid content of 7.72%, water-reduction ratio of 23%, and pH of 4.56 was used. The tap water was used for mixing. The NC mix proportions are given in Table 2.

For UHPC, the same cement and ground granulated blast-furnace slag as those used in NC were adopted, and silica fume (Guangxi Xintumao Technology Co., Ltd., Nanning, China) was added; the relevant properties are shown in Table 1. River sand served as the fine aggregate (apparent density: 2650 kg/m^3^; maximum particle size: 5 mm; fineness modulus: 2.90, China Resources Zhizhu Technology (Nanning) Co., Ltd., Nanning, China). Copper-coated steel fibers (13 mm in length and 0.18 mm in diameter, Hengshui Zhongxiang Engineering Materials Co., Ltd., Hengshui, China) with a tensile strength of 2850 MPa were used. A polycarboxylate-based high-performance water reducer (No. 2, Jiangsu Sobute New Materials Co., Ltd., Nanjing, China) with a solid content of 30%, water-reduction ratio of 35%, and pH of 6.5, was employed. The tap water was used for mixing. The UHPC mix proportions are presented in Table 2.

### 2.2. Specimen Preparation

**Figure 2:**
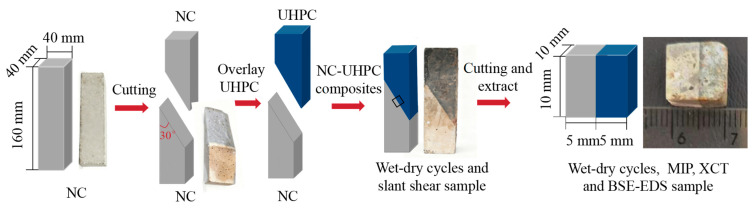
Preparation of NC-UHPC composites for wet–dry cycling, slant shear testing, and sampling for MIP, XCT and BSE-EDS.

The NC-UHPC composite specimens were prepared by casting a UHPC overlay onto precast NC substrates, as illustrated in Figure 2. For NC, sand, cement, ground granulated blast-furnace slag, and limestone powder were dry-mixed for 30 s. Polycarboxylate-based high-performance water reducer (No. 1) was premixed with water, and 4/5 of the solution was added to the mixer and mixed for 90 s. The mortar adhering to the blades and inner wall of the high-speed mixer was scraped back into the main mixture, after which crushed stone and the remaining polycarboxylate-based high-performance water reducer (No. 1) solution were added and mixed for a further 90 s. The fresh NC mixture was cast into 40 mm × 40 mm × 160 mm steel molds and compacted using a cement-based tamping rod to remove entrapped air. After 24 h of curing at 20 ± 5 °C and ≥50% relative humidity, specimens were demolded and cured for 28 days at 20 ± 2 °C and ≥95% relative humidity. Subsequently, the substrate surface was cut using a water-cooled saw to form a 30° bevel, producing a 40 mm × 80 mm contact surface. The cut surface was thoroughly rinsed with water to prepare the UHPC casting interface, and the NC substrate was returned to the mold.

For UHPC, cement, ground granulated blast-furnace slag, silica fume, and sand were dry-mixed for 30 s. Polycarboxylate-based high-performance water reducer (No. 2) was premixed with water and added at once, followed by mixing for 5 min. Steel fibers were then gradually and uniformly introduced during mixing, and mixing was continued for 6 min; fiber addition was completed within 1 min. The fresh UHPC was cast onto the prepared NC substrate in the same steel mold and compacted to remove entrapped air. The resulting NC-UHPC composites were statically cured for 24 h at 20 ± 5 °C and 50% relative humidity, followed by curing for 28 days at 20 ± 2 °C and 95% relative humidity.

For the XCT, MIP and BSE-EDS analyses, part of the 40 mm × 40 mm × 160 mm NC-UHPC specimens was sectioned into 10 mm × 10 mm × 10 mm cubes (Figure 2), each containing the NC, OTZ, and UHPC regions. Six groups of cube specimens were prepared. One group was scanned by XCT before testing (0 cycles) and rescanned after 90 wet–dry cycles, enabling damage tracking in the same specimen. The other five groups were used for MIP and BSE-EDS testing at 0, 30 and 90 cycles, respectively.

### 2.3. Seawater Wet–Dry Cycling Regime

The chloride-ion concentration in seawater from the Longmen Strait (Qinzhou Bay, Guangxi Zhuang Autonomous Region, China) ranges from 10,856.6 to 10,900.9 mg/L, and the sulfate-ion concentration ranges from 1440.9 to 1741.1 mg/L [49]. To accelerate corrosion-induced degradation of NC-UHPC composites, artificial seawater was prepared with chloride and sulfate ion concentrations set to 10 times those of natural seawater. Increasing the ion concentration of the immersion solution is a commonly used accelerated testing strategy in studies of marine concrete durability under laboratory conditions [50]. This elevated ion concentration does not alter the fundamental degradation mechanisms of the composite system, namely chloride-induced steel-fiber corrosion, erosion of cement hydration products by sulfate ions, and pore-structure evolution caused by wet–dry cycles [6,51,52]. Instead, it allows observable damage to develop within a practical laboratory timescale. In general, the degradation rate increases with increasing ion concentration [51,53]. Accordingly, the target chloride and sulfate ion concentrations were 108.8 and 15.9 g/L, respectively. Industrial-grade sodium chloride and magnesium sulfate dihydrate (purity > 98%, China Salt Xingan Salt Chemical Co., Ltd., Ji’an, China) were used to prepare the artificial seawater.

Wet–dry cycling was performed in accordance with GB/T 50082 [54]. Specimens were immersed for 15 h in solution at 20 ± 2 °C (artificial seawater or municipal water, as specified), removed, and dried until the surface was free of visible water, followed by oven drying at 60 ± 1 °C for 9 h. This sequence constituted one cycle. For the 40 mm × 40 mm × 160 mm NC-UHPC composite specimens, the wet–dry cycling was conducted for 0, 30, 60, and 90 cycles, with three parallel specimens in each group. For the 10 mm × 10 mm × 10 mm cube specimens used for XCT and MIP, only the 0 and 90 cycles conditions were considered, as described in Section 2.2, while for the BSE-EDS tests, the 30-cycle condition was additionally included.

### 2.4. Slant Shear Test

Slant shear tests were conducted on 40 mm × 40 mm × 160 mm NC-UHPC composite specimens (three replicates per group) according to the ASTM C882-23 [55]. The testing machine had a maximum load capacity of 300 kN, and loading was applied at 0.1 kN/s. The normal compressive stress (*σ_n_*) and shear bond stress (*τ_n_*) at failure were calculated using Equations (1)–(3) [56,57,58].

(1) $$\sigma_{0}=\frac{F_{u}}{A_{0}}$$

(2) $$\sigma_{n}=\sigma_{0}{\mathrm{Sin}}^{2}\alpha$$

(3) $$\tau_{n}=\frac{1}{2}\sigma_{0}\mathrm{Sin}\left(2\alpha \right)$$

where *F_u_* is the ultimate load, *A*_0_ is the loaded area (1600 mm^2^), *σ*_0_ is the nominal compressive stress, and *α* is the inclination angle of the bonded interface (30°). *σ_n_* and *τ_n_* are the normal and shear bonding stresses acting on the interface induced by *σ*_0_, respectively.

### 2.5. Mass Loss Measurement

To quantify mass changes during corrosion, three NC-UHPC specimens (160 mm × 40 mm × 40 mm) were weighed after 0, 30, 60, and 90 wet–dry cycles. Weighing was performed using a balance with a maximum capacity of 1000 g and an accuracy of 0.01 g. The mass loss rate was calculated using:

(4) $$\omega =\frac{m_{0}-m_{1}}{m_{0}}\times 100\%$$

where *w* is the mass loss rate (%), and *m*_0_ and *m*_1_ are the specimen masses before and after wet–dry cycling, respectively.

### 2.6. Backscattered Electron Imaging Coupled with Energy Dispersive X-Ray Spectroscopy Test

To characterize the effect of wet–dry cycles on the morphology and elemental distribution of NC-UHPC composites, 10 mm × 10 mm × 10 mm cube specimens prepared in Section 2.2 were subjected to BSE-EDS analysis after 0, 30, and 90 cycles, respectively. The NC-UHPC interface was examined using BSE technology. For BSE-EDS analysis, the NC-UHPC composite specimens were prepared according to the procedure described in Section 2.2 and then polished with alumina polishing powder to obtain a smooth and flat surface. Prior to testing, the specimens were dried at 60 ± 5 °C for at least 24 h. BSE analysis was performed using a Sigma360 microscope (Carl Zeiss AG, Oberkochen, Germany) operated at 15 kV, with a magnification range of 100×–500,000×. The EDS analysis was performed using an Oxford Xplore 30 instrument (Oxford Instruments plc, Abingdon, United Kingdom).

### 2.7. Mercury Intrusion Porosimetry Test

To characterize pore-structure changes in NC-UHPC induced by wet–dry cycling, 10 mm × 10 mm × 10 mm cubes prepared in Section 2.2 were tested by MIP after 0 and 90 cycles, using two separate groups of specimens because MIP is destructive. The pore-size distribution was then determined. Pore-size distributions were measured by MIP using an AutoPore IV 9500 porosimeter (Micromeritics Instrument Corporation, Norcross, GA, USA). The instrument has a maximum pressure of 228 MPa and measures pore diameters ranging from 5 to 8 × 10^5^ nm. Prior to testing, specimens were oven-dried at 105 ± 1 °C for 24 h.

### 2.8. X-Ray Computed Tomography

#### 2.8.1. XCT Scanning and Reconstruction

To obtain complementary pore-structure information, the MIP was used to quantify pore-size distribution and porosity, whereas the XCT was employed to characterize pore geometry and morphology [32]. The XCT specimens were 10 mm × 10 mm × 10 mm cubes prepared in Section 2.2. The same group of specimens was scanned before the wet–dry cycling (0 cycles) and rescanned after 90 wet–dry cycles to track the evolution of damage in the same specimen. Scanning was conducted using a NanoVoxel 2000 system (Sanying Precision Instruments Co., Ltd., Tianjin, China) operated at 20 kV and 50 μA. The source-to-sample and sample-to-detector distances were 25.768 mm and 427.948 mm, respectively, with an exposure time of 1 s and a voxel size of 5 μm. A total of 400 slices were reconstructed, with an image matrix of 2400 × 2200 pixels per slice.

**Figure 3:**
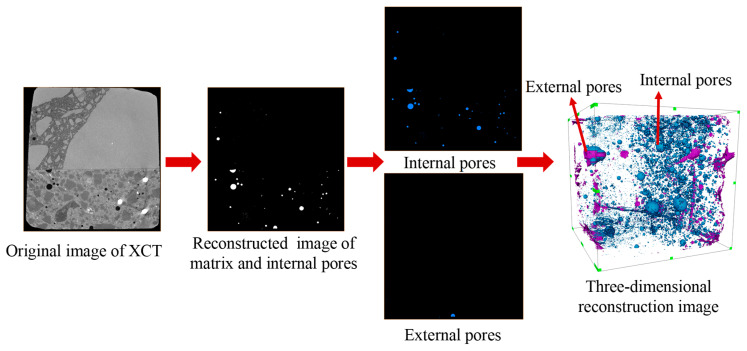
XCT segmentation process.

The XCT segmentation workflow is shown in Figure 3. To improve the accuracy of pore identification after wet–dry cycling, image contrast was first enhanced, followed by Boolean operations to isolate internal pores (the pores marked in blue in Figure 3). An ambient occlusion-based algorithm was then used to distinguish external pores (the pores marked in purple in Figure 3) from the background. This procedure reduces background misclassification and improves the detection accuracy of external pores [32,59,60].

#### 2.8.2. Fractal Dimension Analysis

The fractal theory was initially developed to characterize irregular natural geometries and has since been widely applied in materials science. It enables quantitative analysis of pore deformation and evolution from initiation and propagation to final failure [32,61]. Among the available methods, the box-counting method is widely used due to its simplicity and practicality. In this method, square boxes with different side lengths (*L*) are used to cover the target image, and the corresponding number of boxes, *N*(*L*), is recorded. The fractal dimension is then determined from the relationship between log *N*(*L*) and log (1/*L*) as *L* approaches zero, as expressed in Equation (5) [32,46,47].

(5) F=limL→0logNLlog1L

If *F_i_* denotes the sum of the fractal dimensions of the *i*th slice (*n* = 1, 2, 3, …), the summation expression is given as:


(6) $$F_{i}=\sum_{n=1}^{n}\;F_{n}$$

The fractal dimension reflects the complexity of the internal pore–crack structure. A higher fractal dimension indicates a higher degree of internal damage, more developed cracks, and greater structural complexity [32,46,47].

#### 2.8.3. Porosity Characterization

The porosity was determined based on image-processing results, which distinguished two pore categories within the specimen: externally connected pores and isolated internal pores. This procedure also enables three-dimensional reconstruction of the pore structure before and after wet–dry cycling (Figure 3), where the blue areas represent isolated internal pores and purple areas represent externally connected pores. In this study, the concrete porosity was calculated on the basis of voxel volume [32]:

(7) $$\phi =\frac{V_{ex}+V_{in}}{V_{total}}\times 100\%$$

where *ϕ* is porosity; *V_ex_* is the voxel volume of the externally connected pores; *V_in_* is the voxel volume of the internally isolated pores; *V_total_* is the total voxel volume of the specimen.

## 3. Results

### 3.1. Surface Degradation, Failure Patterns, and Bond Strength Evolution

**Figure 4:**
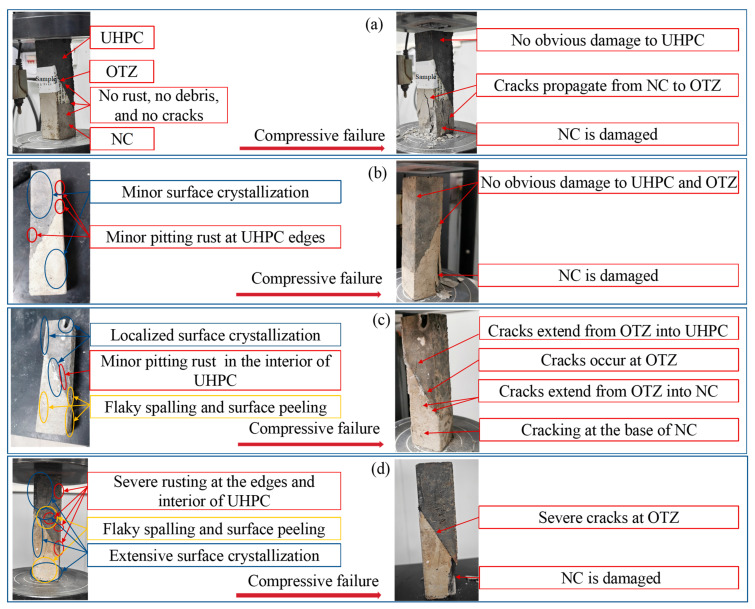
The surface degradation and failure patterns of the specimens at different wet–dry cycling stages: (**a**) 0 wet–dry cycles, (**b**) 30 wet–dry cycles, (**c**) 60 wet–dry cycles and (**d**) 90 wet–dry cycles.

**Figure 5:**
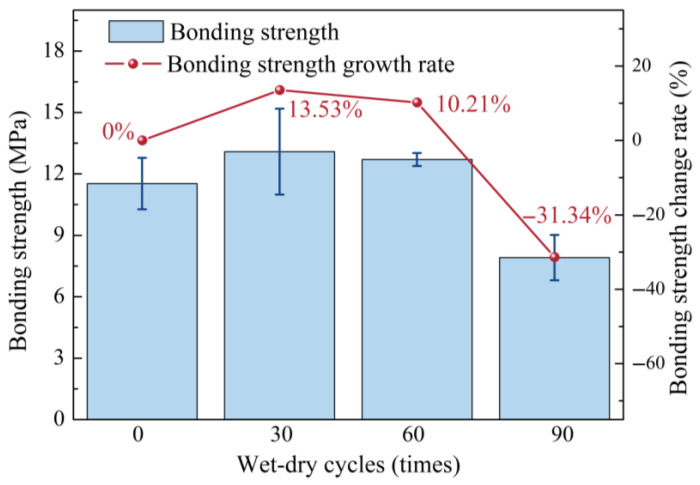
Interfacial bond strength of the NC-UHPC specimens.

Figure 4 displays the surface degradation and failure patterns of the specimens at different wet–dry cycling stages. Figure 5 shows the interfacial bond strength of the specimens. In Figure 5, the bond strength of NC-UHPC composites first increased and then decreased, which is consistent with the strength evolution of concrete under sulfate wet–dry cycles reported in previous studies [53,62,63].

Before the wet–dry cycling, the specimens remained intact, with no visible rust, salt crystallization, or surface cracking (Figure 4a). After the slant shear test, damage was mainly concentrated in the relatively weaker NC region, while no obvious failure was observed in the OTZ or UHPC. This indicates that the initial interfacial bond strength exceeded the strength of the NC substrate.

After 30 wet–dry cycles, a small quantity of salt crystallization appeared on the specimen surface, and localized corrosion was observed near the UHPC edge (Figure 4b). After the slant shear test, failure was still mainly concentrated in the NC region, and the OTZ and UHPC remained largely intact. Compared to the uncycled condition, the bond strength increased by 13.53%. This increase suggests that, at the early stage of exposure, continued hydration and the filling of micropores by hydration products in the NC and OTZ outweighed corrosion-induced deterioration, thereby enhancing interfacial bonding [64].

After 60 wet–dry cycles, surface corrosion became more pronounced, with a larger rusted area and substantial salt crystallization, especially around existing cracks (Figure 4c). Local pitting and peeling were observed in both NC and UHPC. Following the slant shear test, cracks initiated in the OTZ and propagated into both the NC and UHPC, accompanied by cracking of the mortar matrix. The bond strength decreased by 3.32% compared with that after 30 cycles.

After 90 wet–dry cycles, the specimens exhibited severe surface degradation, characterized by extensive rusting, significant salt crystallization, and local pitting and peeling (Figure 4d). Although the overall specimen remained macroscopically intact, the OTZ became the primary failure region after the slant shear test, with additional damage in the NC. The bond strength decreased sharply by 41.55% relative to that after 60 cycles. Overall, with increasing number of wet–dry cycles, the dominant damage region gradually shifted from NC to the OTZ, and the bond strength exhibited an initial increase followed by a marked decline.

### 3.2. XCT Characterization of Surface Damage and Pore Evolution

**Figure 6:**
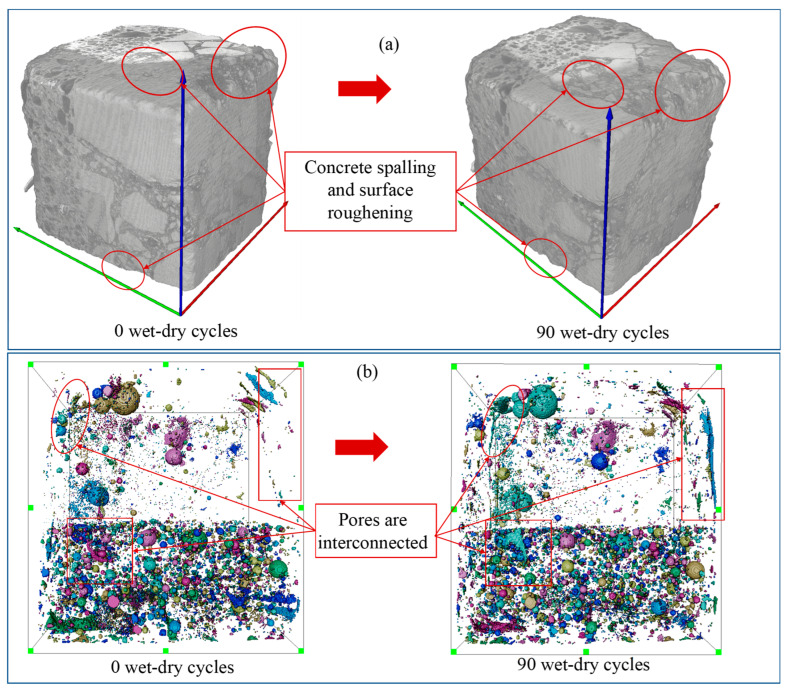
XCT-based 3D reconstruction of the NC-UHPC composites: (**a**) appearance changes; (**b**) pore structure changes.

To further clarify the structural basis for this macroscopic deterioration and bond-strength evolution, the XCT was used to characterize 3D surface damage and pore-network development. Figure 6 presents the XCT-based 3D reconstructions and pore-structure evolution of the NC-UHPC composites before and after wet–dry cycling. As shown in Figure 6a, the specimen surface before cycling was dense and intact. After 90 cycles, pronounced surface roughening and spalling were observed mainly in the NC region, whereas the OTZ and UHPC remained relatively intact, indicating that deterioration was concentrated primarily in the NC region. Figure 6b further shows the evolution of the pore structure, where different colors are used to mark the pores to better distinguish whether the pores are in an isolated and dispersed state or connected to each other. At 0 cycles, pores were relatively sparse and locally distributed. After 90 cycles, the pore network became more dispersed and interconnected, accompanied by an increased proportion of large pores. These observations indicate that wet–dry cycles promoted the initiation, propagation, and coalescence of pore defects [65,66].

### 3.3. Porosity Evolution and Mass Change

**Figure 7:**
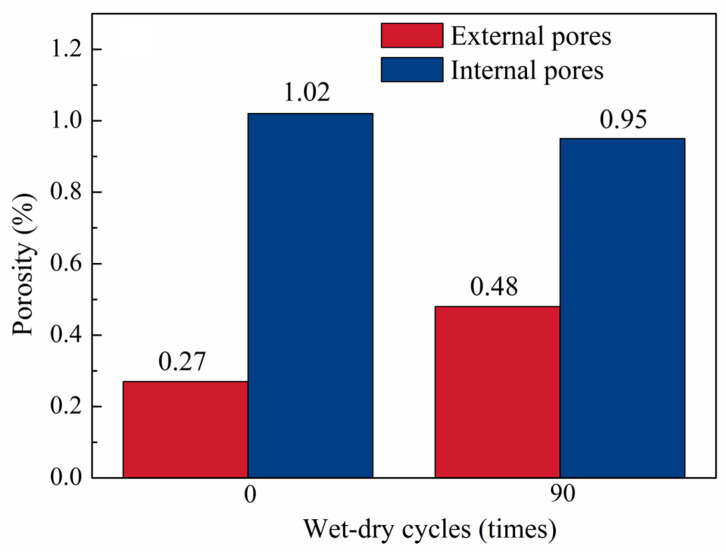
Changes in internal and external porosity.

**Figure 8:**
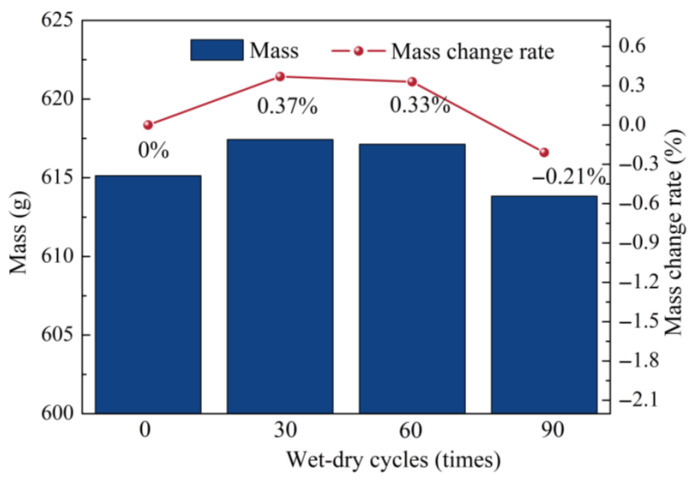
Changes in mass.

Figure 7 and Figure 8 quantify the evolution of porosity and specimen mass during wet–dry cycling. As shown in Figure 7, after 90 cycles, the total porosity increased by 0.14%, with external porosity increasing by 0.21% and internal porosity decreasing by 0.07%, indicating the development of externally connected pores at the expense of isolated internal pores. As shown in Figure 8, the specimen mass increased by 0.37% after 30 cycles, then decreased by 0.04% at 60 cycles relative to that after 30 cycles, and further decreased by 0.54% at 90 cycles relative to that after 60 cycles. This non-monotonic mass evolution is consistent with previous observations for concrete under sulfate wet–dry cycles by Zhou [63] and Yang [53], although the inflection point differed because of the NC-UHPC composite system and the combined chloride–sulfate exposure. Overall, these results reflect progressive surface deterioration and material loss during prolonged wet–dry cycling.

**Figure 9:**
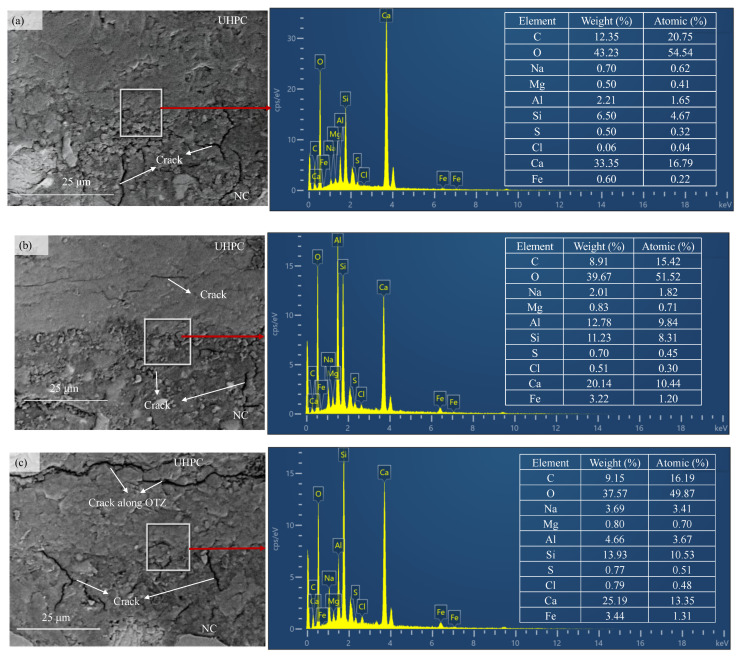
BSE-EDS analysis results of NC-UHPC specimens after different wet–dry cycles: (**a**) 0 wet–dry cycles, (**b**) 30 wet–dry cycles, (**c**) 90 wet–dry cycles.

To further clarify the degradation behavior and mass evolution of the NC-UHPC composites during wet–dry cycling, the distributions of Cl and S and their relationship with mass change were analyzed based on the BSE-EDS surface mapping results shown in Figure 9.

As indicated by the quantitative EDS results, the contents of Cl and S exhibited a clear increasing trend with increasing number of the wet–dry cycles. At 0 cycles, the mass fractions of Cl and S were 0.50% and 0.06%, and their atomic fractions were 0.04% and 0.32%, respectively, representing only the background levels introduced by impurities in the raw materials. After 30 wet–dry cycles, the mass fractions of Cl and S increased to 0.51% and 0.70%, while the corresponding atomic fractions increased to 0.30% and 0.45%, respectively. These results indicate that Cl^−^ and SO_4_^2−^ penetrated inward through the initial cracks in the NC matrix, and that ions ingress and their interaction with hydration products were closely associated with the initial increase in specimen mass. After 90 cycles, the mass fractions of Cl and S further increased to 0.79% and 0.77%, with atomic fractions of 0.48% and 0.51%, respectively. Meanwhile, through-cracks extending along the interface were observed in the OTZ region (Figure 9c). Continued ingress of Cl^−^ and SO_4_^2−^ promoted the formation of expansive products, such as ettringite and Friedel’s salt, which induced internal stress concentration and matrix spalling, thereby contributing to the mass decrease observed at the later stage of cycling [6,67,68].

Combined with the BSE micromorphology, these results show that the penetration paths of Cl^−^ and SO_4_^2−^ during wet–dry cycling were closely associated with the initial cracks and defects in the NC matrix. In the early stage of cycling, ions preferentially migrated through the surface cracks on the NC side toward the OTZ, where adsorption and hydration-related reactions occurred, corresponding to the initial mass increase of the specimen. In the middle and later stages, the volumetric expansion caused by newly formed products intensified crack propagation in the OTZ, and eventually led to spalling of the concrete surface layer, causing the specimen mass to shift from increase to decrease. The continuous increase in Cl and S contents in the EDS results further supports the role of ion ingress in the observed mass evolution [6].

### 3.4. XCT-Observed Local Damage Evolution

**Figure 10:**
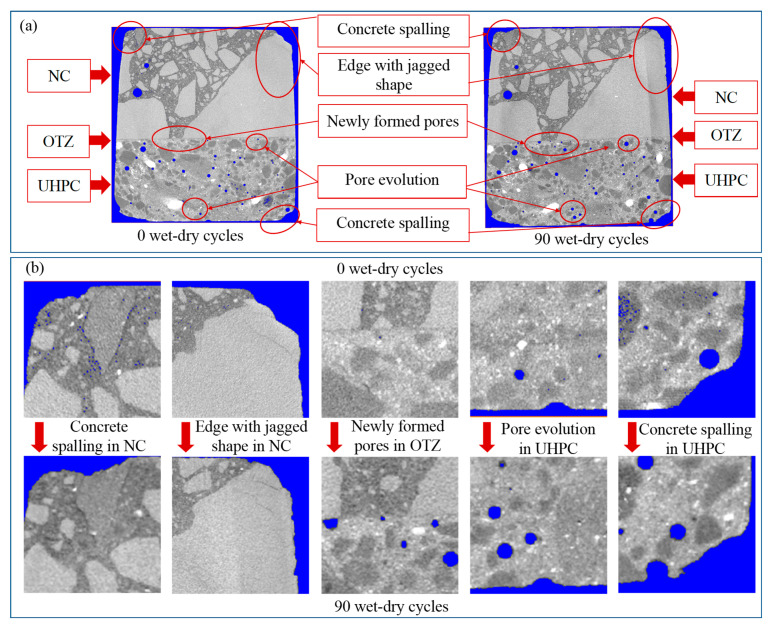
Local damage evolution of the NC-UHPC composites at the same position after 0 and 90 wet–dry cycles: (**a**) overall slice view, (**b**) magnified local view.

Figure 10 shows the comparison of the local damage evolution of the NC-UHPC composite at the same position after 0 and 90 wet–dry cycles. In the reconstructed images, the gray region represents the concrete matrix, the OTZ is indicated by the red arrows, the upper part corresponds to NC, the lower part to UHPC, the white regions denote steel fibers, and the blue regions represent pores. After 90 wet–dry cycles, the composite exhibited pronounced local deterioration. The NC edge showed a serrated corrosion morphology accompanied by slight spalling and microcracking, indicating reduced surface integrity. In the OTZ and UHPC regions, both pore number and pore size increased, and the local structure became visibly looser. Slight edge spalling and more pronounced external pores were also observed in UHPC, although no obvious cracks were detected. Overall, the quantified damage evolution across the NC/OTZ/UHPC three-phase region shows that damage was most severe in NC, followed by OTZ, and least severe in UHPC.

### 3.5. Slice-Wise Porosity Evolution

**Figure 11:**
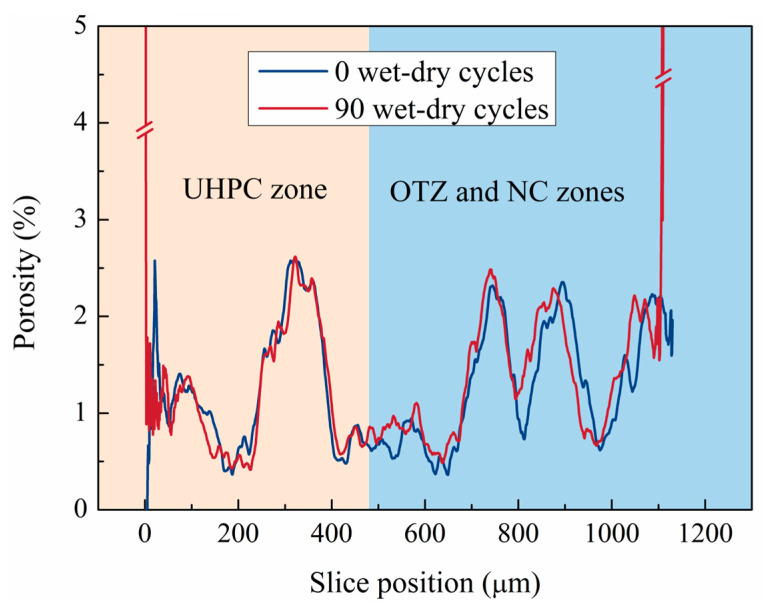
Slice-wise porosity distribution across the NC/OTZ/UHPC three-phase region.

Figure 11 shows the slice-wise porosity distribution across the NC/OTZ/UHPC three-phase region. Along the coordinate axis, the UHPC region is located within 0–485 μm, while the OTZ and NC regions correspond to 486–1130 μm. The porosity in the NC region exhibited significant fluctuations. Porosity troughs mainly corresponded to slice passing through the aggregate-rich regions, where the aggregates were denser than the surrounding cement matrix, whereas porosity peaks were mainly associated with the more porous mortar matrix. These local differences in aggregate–matrix distribution were the main reason for the observed porosity fluctuations.

After the wet–dry cycling, porosity increased markedly near the NC-UHPC interface, and the porosity in the OTZ and NC regions also increased significantly. The non-uniform evolution of porosity peaks after 90 cycles, with some increasing and others decreasing, reflected the competing effects of pore filling and pore coarsening during seawater wet–dry cycling. On one hand, continued hydration of unhydrated cement particles and the formation of expansive products, such as ettringite and Friedel’s salt, filled fine pores and refined the local pore structure [6,67,68]. On the other hand, long-term ion erosion, micropore coalescence, and microcrack propagation progressively damaged the matrix and promoted pore coarsening, leading to the development of larger pores [69,70]. As a result, porosity peaks at different pore sizes exhibited different evolution trends rather than a uniform change pattern. By contrast, the internal porosity of UHPC decreased slightly, consistent with the results in Figure 7. This reduction may be attributed to the limited ingress of salt solution into the dense UHPC matrix, where newly formed products partially filled the original pores and increased local compactness [53]. Overall, these results indicate pronounced spatial heterogeneity in pore evolution across the NC/OTZ/UHPC three-phase region.

### 3.6. Fractal Dimension and Pore Connectivity

**Figure 12:**
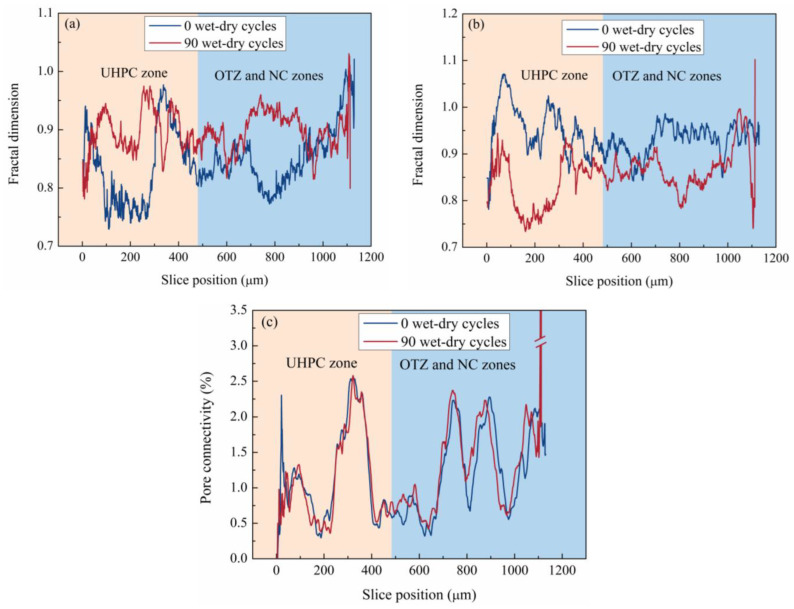
Evolution of pore complexity and connectivity: (**a**) internal pore fractal dimension, (**b**) external pore fractal dimension, (**c**) pore connectivity.

The evolution of pore complexity and connectivity during wet–dry cycling is further characterized in Figure 12. As shown in Figure 12a,b, the internal pore fractal dimension increased noticeably in the UHPC, OTZ, and NC regions, whereas the external pore fractal dimension decreased. The increase in internal fractal dimension indicates that the internal pore–crack system became more heterogeneous and complex after the wet–dry cycling. In contrast, the decrease in external fractal dimension suggests that externally connected pores became more open and simplified as surface damage and pore coalescence progressed. Within the 300–350 μm range, where steel fibers were concentrated, the internal pore fractal dimension after the wet–dry cycling was lower than that before cycling. This behavior may be related to crack development in the fiber–matrix interfacial transition zone caused by local deformation mismatch.

Figure 12c further shows the evolution of pore connectivity. Near 350 μm, where steel fibers were concentrated, both pore connectivity and porosity increased, indicating the formation of interfacial defects in the fiber–matrix transition zone. In the 480–800 μm range, close to the OTZ on the NC side, pore connectivity increased significantly after wet–dry cycling, which is consistent with crack propagation from NC toward the OTZ. These results suggest that crack development plays a dominant role in enhancing pore connectivity.

### 3.7. Multi-Scale Pore-Size Distribution from XCT and MIP

**Figure 13:**
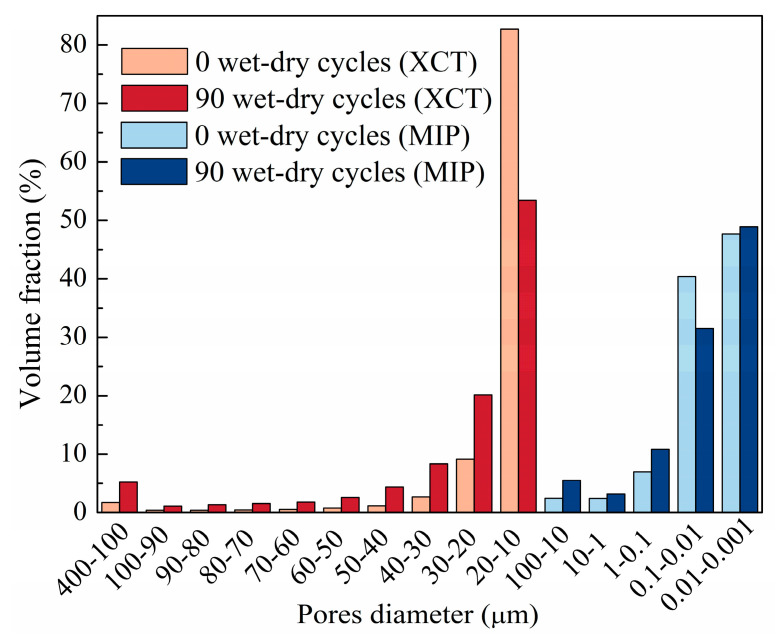
Multi-scale pore-size distributions obtained from XCT and MIP.

Figure 13 presents the multi-scale pore-size distributions obtained from XCT and MIP. For pores larger than 10 μm characterized by XCT, the pore volume fraction at 0 wet–dry cycles was dominated by the 10–20 μm range, accounting for 82.70%, indicating that the initial larger pores were mainly relatively isolated pores of medium size. After 90 wet–dry cycles, the proportion of pores in the 10–20 μm range markedly decreased to 53.43%, whereas that in the 20–50 μm range increased from 13.04% to 32.86%. In addition, the proportion of pores in the 100–400 μm range increased from 1.75% to 5.22%. These changes indicate pronounced pore coarsening, accompanied by pore expansion, coalescence, and development of microcracks that promoted the interconnection of adjacent pores. For pores smaller than 10 μm characterized by MIP, the 0.001–0.1 μm range accounted for 88.12% at 0 wet–dry cycles, whereas the fractions in the 0.1–1 μm and 1–10 μm ranges were relatively low. After 90 wet–dry cycles, the proportion of pores in the 0.001–0.1 μm range decreased to 80.41%, while those in the 0.1–1 μm and 1–10 μm ranges increased significantly. This shift indicates a redistribution of the pore system from finer pores towards the larger pores, suggesting micropore coalescence and pore enlargement during wet–dry cycling. Overall, the combined XCT and MIP results demonstrate that wet–dry cycling progressively transformed the pore system from relatively isolated fine pores to a more connected and coarser pore network, thereby providing the structural basis for the observed deterioration in interfacial bond strength.

## 4. Discussion

### Microstructural Damage Evolution Mechanism of NC-UHPC Composites

**Figure 14:**
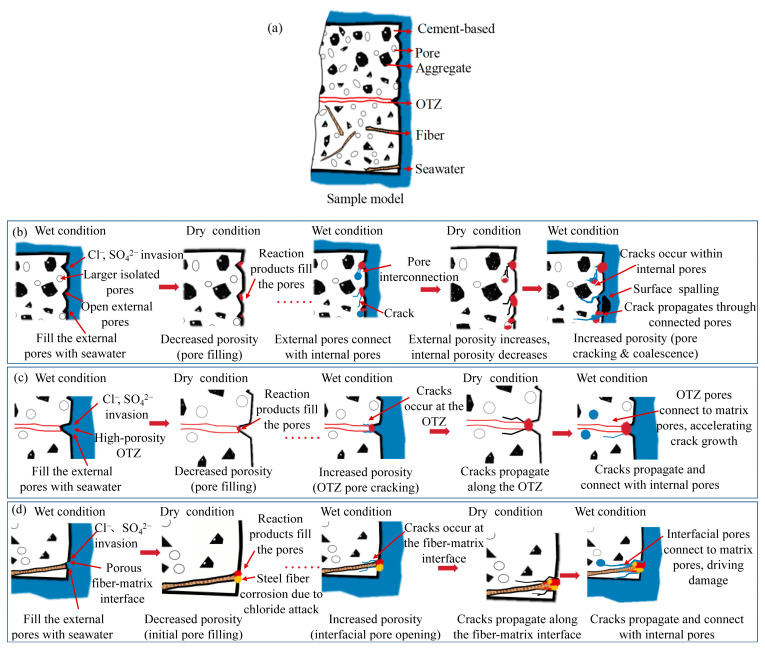
Microstructural damage evolution of the NC-UHPC composites: (**a**) schematic of the sample model, (**b**) damage evolution in NC, (**c**) damage evolution in OTZ and (**d**) damage evolution in UHPC.

Figure 14 illustrates the microstructural damage evolution of NC-UHPC composites under the seawater wet–dry cycles. The ellipsis “……” in Figure 14b represents the process of undergoing several wet-dry cycles. Although the deterioration develops simultaneously in the NC, OTZ, and UHPC regions, its severity is clearly region-dependent, being highest in NC, followed by OTZ, and lowest in UHPC, which is fully consistent with the surface observations, XCT results, and interfacial bond-strength evolution. During the wetting stage, seawater rapidly enters externally connected pores and further penetrates the interior through capillary suction. Chloride ions promote steel-fiber corrosion [71], while sulfate ions react with cement hydration products to form expansive reaction products, such as ettringite and gypsum [72,73,74]. During drying, water evaporation increases the ion concentration within pores and promotes salt crystallization. These crystalline salts can partially fill pores but they generate crystallization pressure on the pore walls, as well thereby inducing local cracking and pore enlargement. Through repeated wetting, drying, dissolution, and recrystallization, wet–dry cycling progressively aggravates pore damage, crack propagation, and structural deterioration [37,38,39].

The deterioration of NC initiates the earliest since its relatively high porosity facilitates rapid seawater ingress. In the early stage of exposure, externally connected pores first interact with the salt solution, which explains the increase in external porosity and the decrease in the external pore fractal dimension observed in the results. At the same time, part of the internal pore system is temporarily filled by newly formed products, leading to a slight reduction in internal porosity, increased internal pore complexity, and a short-term mass increase. As wet–dry cycling continues, corrosive ions penetrate deeper into the NC matrix, and the combined action of crystallization pressure, expansive reaction products, and cyclic fatigue promotes pore coalescence and crack interconnection. This interpretation agrees with the XCT and MIP results showing a transition from relatively isolated fine pores to a coarser and more connected pore system. Once this process intensifies, the NC surface becomes loose and vulnerable to spalling, which explains the later-stage mass loss and severe local deterioration observed in the results.

Compared to NC, the OTZ shows better resistance at the early stage of wet–dry cycling, although it remains the weakest structural link in the composite system. This behavior is associated with its relatively high initial porosity, which provides limited buffering space for crystallization pressure and delays the onset of severe cracking. In addition, the dense UHPC side restricts the transport of chloride and sulfate ions, so early damage in the OTZ is mainly concentrated near the NC side. This explains why the initial failure after the slant shear was still dominated by the NC region and why the interfacial bond strength increased after 30 cycles. However, with continued wet–dry cycling, the OTZ is subjected to long-term deformation mismatch and uncoordinated stress transfer between NC and UHPC. As a result, interfacial microcracks nucleate and propagate, which is consistent with the XCT-observed increase in porosity and connectivity near the OTZ and with the shift of the dominant failure region from NC toward the OTZ. Once these cracks coalesce, the OTZ becomes the primary damage zone under loading, leading to synchronous failure with the adjacent NC and a pronounced reduction in interfacial bond strength after prolonged exposure.

The UHPC exhibits the highest resistance to wet–dry cycling because of its dense matrix and the bridging effect of steel fibers. In the early stage, although the external surface of UHPC is directly exposed to seawater, the compact microstructure limits deformation and crack growth caused by crystallization pressure. This explains why the internal porosity and pore connectivity of UHPC change only slightly and may even decrease locally, while the internal pore fractal dimension increases because the remaining pore system becomes more heterogeneous at a finer scale. However, with an increasing number of wet–dry cycles, corrosion of the steel fibers gradually exacerbates local surface deterioration in the NC-UHPC composites. The resulting defects around the fiber–matrix interfacial transition zone provide additional transport pathways for aggressive ions, which is consistent with the local increase in porosity and connectivity in the fiber-concentrated region. Even so, the overall damage in UHPC remains substantially lower than that in NC and OTZ, and obvious through-cracking is largely suppressed.

The evolution of interfacial bond strength can therefore be interpreted as the combined result of early-stage densification and later-stage degradation. At low numbers of wet–dry cycles, continued hydration and partial pore filling improve the compactness of the NC and OTZ regions, which explains the initial increase in bond strength. As exposure continues, however, the beneficial effect of densification is progressively overtaken by salt crystallization, expansive reaction products, steel-fiber corrosion, and crack coalescence. This transition is directly supported by the XCT and MIP results, which show decreased fractions of fine pores, increased fractions of larger pores, higher external porosity, and enhanced pore connectivity after prolonged cycling. Once the OTZ becomes highly connected and structurally weakened, it can no longer effectively transfer load across the interface, resulting in the sharp decrease in bond strength observed after 90 wet–dry cycles. Overall, the deterioration of NC-UHPC composites under seawater wet–dry cycling is governed by the coupled effects of ion ingress, crystallization pressure, expansive chemical reactions, and region-dependent pore evolution. The combined macroscopic and multi-scale microstructural results consistently demonstrate that damage initiates in NC, develops toward the OTZ, and remains least pronounced in UHPC. This region-dependent damage sequence provides the structural explanation for the observed transition from early-stage strengthening to later-stage interfacial degradation. However, it should be noted that the present study was conducted under an accelerated and simplified laboratory exposure condition. Therefore, the present results should be interpreted primarily as a mechanistic and comparative understanding of NC-UHPC deterioration under seawater wet–dry cycling, rather than as a direct quantitative representation of field degradation in real marine environments. Nevertheless, the combined XCT-MIP-slant shear approach may also provide a useful framework for investigating seawater-induced deterioration in other cement-based composites with distinct interface regions.

## 5. Conclusions

In this study, the deterioration behavior of NC-UHPC composites under seawater wet–dry cycling was systematically investigated through the combined use of XCT, MIP, and macroscopic mechanical tests. The main conclusions are as follows:

(1) Interfacial bond strength exhibited a non-monotonic evolution under wet–dry cycling. The bond strength of the NC-UHPC composites first increased and then decreased with increasing cycle number. Specifically, it increased by 13.53% after 30 cycles and then decreased sharply by 41.55% after 90 cycles relative to that after 60 cycles.
(2) Damage evolution in the NC/OTZ/UHPC three-phase region was strongly region-dependent. The damage severity was the highest in NC, intermediate in OTZ, and lowest in UHPC. The NC deteriorated first, showing surface spalling, microcrack initiation, and increased porosity connectivity. In the early stage, the gas-rich pore region in the OTZ exhibited a certain stress-buffering effect and thus showed better damage resistance than NC. With prolonged wet–dry cycling, however, deformation incompatibility between NC and UHPC promoted rapid crack development in the OTZ, causing its damage level to approach that of NC. UHPC maintained the highest resistance, with deterioration mainly limited to surface rusting and an increase in external pores.
(3) The wet–dry cycling led to a clear reconstruction of the pore system. After 90 cycles, the total porosity increased by 0.14%, with external porosity increasing by 0.21% and internal porosity decreasing by 0.07%. Meanwhile, the pore-size distribution shifted from finer pores toward larger pores: the fraction of pores in the 0.001–0.1 μm range decreased from 88.12% to 80.41%, and the fraction of pores in the 10–20 μm range decreased from 82.70% to 53.43%, whereas the proportions of pores in the 20–50 μm and 100–400 μm ranges increased from 13.04% and 1.75% to 32.86% and 5.22%, respectively. These results indicate pore coarsening, pore coalescence, and enhanced connectivity during prolonged exposure.
(4) Fractal-dimension and connectivity analyses further revealed the evolution of internal and external pore structures. With increasing wet–dry cycles, the fractal dimension of internal pores increased, whereas that of external pores decreased. This indicates that the internal pore system became more heterogeneous and complex, while the external pore network became more open and simplified owing to pore expansion and interconnection. The increase in pore connectivity was mainly associated with crack propagation from NC toward the OTZ, while local connectivity enhancement near steel fibers was related to interfacial incompatibility in deformation.
(5) The combined XCT-MIP-slant shear framework proved effective for characterizing seawater wet–dry deterioration in the NC-UHPC composites. The XCT enables non-destructive tracking of damage evolution in the same specimen, while MIP complements XCT by quantifying microscale pore-size redistribution. Together with fractal-dimension analysis and slant shear testing, this framework provides a useful multi-scale basis for evaluating interfacial deterioration in cement-based composites with distinct interface regions and offers practical guidance for the durability design and repair of marine concrete structures.

## Figures and Tables

**Table 1:** Chemical composition and physical properties of the cement, ground granulated blast-furnace slag, limestone powder and silica fume.

Materials		Cement	Ground Granulated Blast-Furnace Slag	Limestone Powder	Silica Fume
Chemical composition (mass%)	SiO_2_	23.14	26.56	4.69	80.96
	Al_2_O_3_	4.37	16.36	1.01	1.89
	Fe_2_O_3_	4.03	0.52	0.47	2.4
	CaO	63.69	34.99	51.94	1.51
	MgO	2.71	10.57	1.17	4.07
	SO_3_	0.24	0.49	0.39	0.26
	f-CaO	0.46	/	/	/
	K_2_O + Na_2_O	0.43	0.13	0.10	0.27
Activity index (%)	7 days	/	85	60	109
	28 days	/	98	61	112
Compressive strength (MPa)	3 days	37.5	/	/	/
	28 days	65.4	/	/	/
Flexural strength (MPa)	3 days	6.7	/	/	/
	28 days	8.7	/	/	/

**Table 2:** Mix proportions of NC and UHPC (kg/m^3^).

	Cement	Water	Ground Granulated Blast-Furnace Slag	Limestone Powder	Silica Fume	Sand	Coarse Aggregate	Steel Fiber	Polycarboxylate-Based High-Performance Water Reducer (No. 1)	Polycarboxylate-Based High-Performance Water Reducer (No. 2)
NC	192	163	134	92	/	868	940	/	10.9	/
UHPC	833	210	200	/	130	1042	/	153	/	31

## Data Availability

The original contributions presented in this study are included in the article. Further inquiries can be directed to the corresponding author.

## Nomenclature

The following nomenclature is used in this manuscript:

NC	Normal concrete
UHPC	Ultra-high performance concrete
OTZ	Overlay transition zone
XCT	X-ray computed tomography
BSE-EDS	Backscattered electron imaging coupled with energy dispersive X-ray spectroscopy
MIP	Mercury intrusion porosimetry
*σ_n_*	Normal compressive stress
*τ_n_*	Shear bond stress
*σ*_0_	Nominal compressive stress
*F_u_*	Ultimate load
*A*_0_	Loaded area
*α*	Inclination angle
*w*	Mass loss rate
*m*_0_	Specimen masses before wet–dry cycling
*m*_1_	Specimen masses after wet–dry cycling
*F_i_*	The sum of the fractal dimensions
*i*	Slice number
*L*	Side lengths of square boxes
*N*	Number of boxes
*ϕ*	Porosity
*V_ex_*	Voxel volume of the externally connected pores
*V_in_*	Voxel volume of the internally isolated pores
*V_total_*	Total voxel volume
